# 94 Weekly participation and inclusion of children using wheelchairs in organized physical activity – an explorative interview study

**DOI:** 10.1093/eurpub/ckae114.121

**Published:** 2024-09-26

**Authors:** Selina Seemüller, Franziska Beck, Frederik Bükers, Claus Krieger, Anne Kerstin Reimers

**Affiliations:** Department of Sport Science and Sport, Friedrich-Alexander-Universität Erlangen-Nürnberg, Erlangen, Germany; Department of Sport Science and Sport, Friedrich-Alexander-Universität Erlangen-Nürnberg, Erlangen, Germany; Faculty of Education, Unit for Movement, Games and Sports Education, University of Hamburg, Hamburg, Germany; Faculty of Education, Unit for Movement, Games and Sports Education, University of Hamburg, Hamburg, Germany; Department of Sport Science and Sport, Friedrich-Alexander-Universität Erlangen-Nürnberg, Erlangen, Germany

## Abstract

**Purpose:**

Organized physical activity (OPA) has great impact to increase social inclusion (Vandermeerschen et al., 2015). Due to its regularity, it can make a great contribution to fulfil the physical activity (PA) recommendations. Nevertheless, less is known about OPA in children using wheelchairs. Therefore, the present study aimed to get a deeper understanding of OPA and possible influencing factors in children using wheelchairs.

**Methods:**

In summary, seven boys and five girls aged 7-11 (mean: 9.67 ± 1.49) years participated in the present qualitative interview study. Using a semi-structured interview guideline, participants were asked about their weekly activities. The interviews analysed OPA type, duration, setting and intensity for each activity. Further, to identify influencing factors of participants’ OPA behavior, a qualitative content analysis with an inductive approach was performed (Kuckartz, 2018).

**Results:**

The following settings were identified across OPA: (club) sport programs, physical education and therapy. The children revealed a mean duration of 166.9 minutes (SD ± 59.7) in moderate to vigorous intensity per week across OPA. Thereby, a lack of wheelchair-sport opportunities as well as a lack of accessibility were identified as main barriers. Due to a lack of competence of teachers and coaches regarding dealing with children using wheelchairs, participants reported of moderate practiced inclusion at school as well as difficulties to participate in non-inclusive settings.

**Conclusions:**

As conclusion, the participants cover only 39.7% of the PA recommendation with OPA. For better inclusion, coaches and teachers need more expertise in dealing with children using wheelchairs. Further, public buildings and schools have to be more accessible and offers of inclusive sports programmes for children using wheelchairs should be increased. The setting therapy is an additional source of PA which should be examined more closely in future studies.

**Support/Funding Source:**

This research did not receive any specific grant from funding agencies in the public, commercial, or not-for-profit sectors.

**References:**

Kuckartz, U. (2018). Qualitative Inhaltsanalyse. Methoden, Praxis, Computerunterstützung. 69 469 Weinheim: Beltz Verlagsgruppe.

Vandermeerschen, H., Vos, S., & Scheerder, J. (2015). Who’s joining the club? Participation of socially vulnerable children and adolescents in club-organised sports. Sport, Education and Society, 20(8), 941-958. doi:10.1080/13573322.2013.856293

